# Metacognitive training: a useful complement to community-based rehabilitation for schizophrenia patients in China

**DOI:** 10.1186/s12888-021-03039-y

**Published:** 2021-01-13

**Authors:** Qi Chen, Yueyun Sang, Lifang Ren, Jinping Wu, Yajun Chen, Menglei Zheng, Guolin Bian, Hanying Sun

**Affiliations:** 1 Department of Chronic Disease and Mental Health, YinZhou Center for Disease Control and Prevention, Ningbo, China; 2 Ningbo Mental Health Center, Ningbo Kangning Hospital, Ningbo, China; 3 Department of Community Health, Hengxi Community Health Center, Ningbo, China; 4 Department of Community Health, Maoshan Community Health Center, Ningbo, China; 5 Department of Community Health, Shounan Community Health Center, Ningbo, China; 6 LKS Faculty of Medicine, The University of Hong Kong, Hong Kong, China

**Keywords:** Schizophrenia, Metacognition, General practitioner, Community rehabilitation

## Abstract

**Background:**

The traditional general practitioner-based model (community-based rehabilitation [CBR]) for Chinese schizophrenia patients lacks sufficient content, usefulness, and theoretical basis for rehabilitation. Based on previous research, we postulate that Metacognitive Training (MCT) is effective in the community for schizophrenic patients.

**Method:**

A randomized controlled, assessor-blinded trial was conducted. A total of 124 schizophrenia patients were recruited from Ningbo China and were randomly assigned to an intervention or a control group. A general practitioner (GP) training plan was carried out before intervention. Intervention and control groups received two CBR follow-ups once a month, while the intervention group, received an additional eight once-a-in-week session of MCT. The Positive and Negative Syndrome Scale (PANSS), and the Psychotic Symptom Rating Scales (PSYRATS) were the primary outcome instruments, while the Quality of Life Scale (SQLS) was the secondary outcome instrument.

**Results:**

In the post-treatment between-groups assessment, the patients in the intervention group showed significantly more reductions on PSYRATS delusions, PSYRATS total, PANSS P6, PANSS core delusions, PANSS positive, PANSS negative, PANSS general and PANSS total, and a significant improvement in SQLS psychosocial aspect.

**Conclusions:**

The study provides preliminary evidence for the usefulness of MCT as a complementary measure for community-based rehabilitation of schizophrenia patients.

**Trial registration:**

ISRCTN, ISRCTN17333276. Registered 09 August 2020 - Retrospectively registered.

## Background

### Psychiatric disorders in the community: current challenges in China

Schizophrenia is a serious and highly disabling psychiatric disorder [1]. In China, the prevalence of psychotic disorders is 1.0% (0.8–1.1) [2], while the point prevalence of schizophrenia in urban areas is 0.68% (rural 0.35%) [3]. Over 90% of schizophrenia patients in China live with their families (in the community) rather than in a psychiatric institution [4]. There is therefore continuous demand for mental health and development (MHD) services at the community level [5].

### Rehabilitation: from hospital-based to community-based

Efforts to improve rehabilitation services have intensified recently in China. For an extended period, the MHD system in China has been hospital-based. Psychiatric physicians conduct short-term rehabilitation in the hospital and follow the discharged patients via phone calls. However, due to various constraints, many of the resources in the hospitals are spent on inpatient treatments, and are not available to discharged patients. Notably, patients have little trust on community health centers and tend to seek health care at larger psychiatric hospitals [6]. However, distance prevents them from receiving hospital-based rehabilitation in several regions.

Hence, the burden of rehabilitation ultimately falls on family caregivers for patients with long-term illness [7]. Though the social burden will largely remain within the family, a supportive community-based rehabilitation (CBR) plan should be made available to help the affected families successfully care for mentally ill members [4]. In China, efforts by the government to shift the focus from hospital-based to community-based yielded plausible results [8], however, CBR services are underutilized [9].

CBR programs for mental health are weakly attached to the primary health care system in China as in other developing counties [5], and are mainly based on a three-tier system. At the municipal level, psychiatric hospital design rehabilitation solutions and pass the patient file to the district level, usually the District Center for Disease Control and Prevention (CDC). The District CDC is responsible for organizing and coordinating various resources. Patients are then assigned to a community health center (community rehabilitation), usually provided by the general practitioner (GP) at the community health center whose work generally includes follow-up and health education, such as recording the patient’s medication, symptoms, and side effects, helping patients complete their rehabilitation plan and giving lectures on mental illness. About 20 to 40 patients are assigned to a GP. Nonetheless, most patients do not have a strong sense of “being taken care of” and consider the follow-up “dispensable” and the health education “boring”. Moreover, the GPs need theoretical-based interventions for community rehabilitation.

### Finding a theoretical-based intervention

There are two main goals in the CBR for mental health: supervision and rehabilitation. The latter aims to decrease psychiatric symptomatology [10]. Delusion is one of these symptoms considered to be significantly correlated with violent acts among psychiatric patients [11]. The challenge for the community is that patients with symptoms of delusion may sometimes cause engage in dangerous behaviors including assaulting others or even suicide [12] and, thus, have some severe consequences in the community.

It is believed that programs that combine active rehabilitation and medications achieve better outcomes as compared to medications alone [13]. Moritz and Woodward developed Metacognitive training (MCT) as an intervention for patients with schizophrenia [14]. Recent studies [15–19] have confirmed that MCT is exciting and can effectively change the patient’s delusional ideation [20]. The efficacy of MCT [21] in patients with schizophrenia spectrum disorder has been reported in several randomized controlled studies. Some of them showed the promising results in terms of immediate posttreatment effect [22–24] and long-term positive psychotic symptoms [16, 25–27]. Recent meta-analyses showed that the MCT can effectively improve the experience of delusions in schizophrenia patients with a small to moderate effect [28, 29]. The primary outcomes of MCT, such as a decline in positive symptoms [30], would meet the requirement of community rehabilitation for the chronic schizophrenic patients [31].

Overall, we aimed at determining whether implementation of MCT is effective under limited conditions such as trainer, recruitment, and location of courses, etc. at the community level.

### About this study

The present study aimed to compare the outcomes of a combined intervention consisting of MCT and CBR with the control group receiving CBR only. The study goes further to confirm the superiority of MCT over CBR for the improvement of delusion.

Studies show that low quality of life generally associates with psychotic symptoms and comorbidities [32], cognitive impairment [33], social isolation, lack of access to environmental resources, and stigmatization of the illness [34].

Some studies have found that severe illness insight may lead to low quality of life [1, 35]. Schizophrenia patients with better insight can realistically evaluate their life and be aware of the enormous negative impact of their illness on their life conditions [36]. Given that illness insight is a target of the MCT [24], which means illness insight would be normally improved in the MCT course, we investigated whether MCT will decrease the quality of life. We also aimed to understand the changes of quality of life among schizophrenic patients pre- and post-intervention.

Finally, we expected to find the evidence of MCT feasibility on community rehabilitation, and for the government to tailor the community services by taking the MCT as a regular complement strategy to the CBR.

## Method

### Recruitment

The trial was conducted at the Yinzhou District of Ningbo, China, with a population size of 1.294 million and a land area of 799.09 km^2^. There were 5262 registered psychiatric patients in the community in the Yinzhou Electronic Healthcare System (EHS) [37], of which 2726 had schizophrenia with a male-to-female sex ratio of 0.75:1 and an average age of 54.69 (12.14).

We first searched for all 2726 schizophrenic patients in the community who met the criteria in the Yinzhou EHS. A total of 1221 patients were excluded due to incomplete electronic records. The primary missing data in the EHS was the PANNS score. Recruitment was then conducted by a psychiatrist from Ningbo Mental Health Center who was assisted by three local GPs. A total of 713 patients had no interest in our project, 428 were too far from the training site and 240 did not meet the criteria. All EHS case data of 124 finally recruited patients were thoroughly reviewed before baseline investigation.

### Design

We designed the study as a randomized controlled, assessor-blinded trial, considering some pragmatic aspects (such as flexibility in intervention delivery) to ensure generalizability of results in the community. The inclusion criteria were age 18–65, a diagnosis of schizophrenia in DSM-IV [38], and a total PANNS score between 50 and 120. Exclusion criteria included psychoactive substances and substance abuse over the last 6 months.

**Fig. 1:**
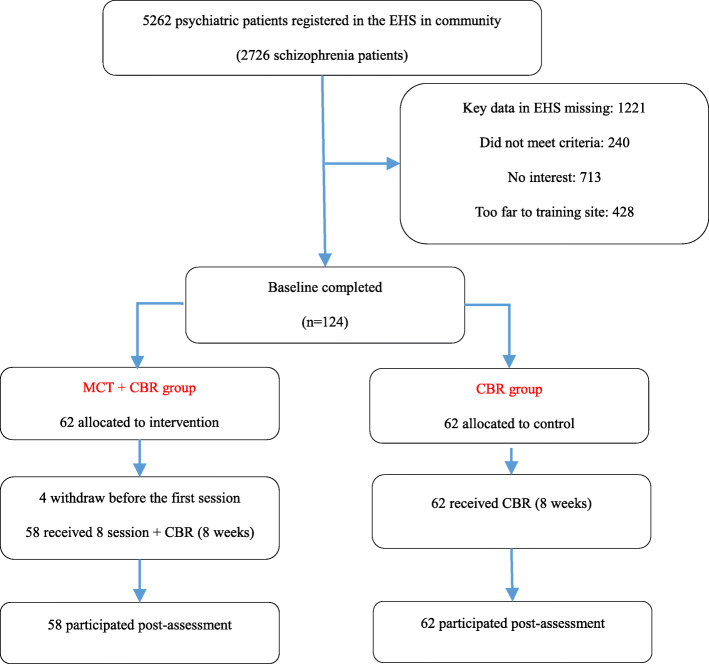
Flow chart

We established three intervention sites based on geographic location. After recruitment, patients were further divided into three groups based on their address. Patients in each group were then randomly assigned to the intervention or control group. Caregivers or patients in the intervention group were informed about the location and the schedule by independent community staff. To ensure safety, we requested a caregiver or a local community staff to accompany each patient during training. The caregiver or local staff stayed in a different room while the patient underwent training. The study was approved by the Ethics Committee of Health Commission of Ningbo (2016C05). All participants provided informed consent to participate in the study. The screening-to-inclusion ratio was 8.2% (see Fig. 1).

### Intervention

The intervention group received CBR plus Metacognitive Training (MCT)^21^, Chinese ver.6.2 (see https://clinical-neuropsychology.de/metakognitives_training_psychose/). The MCT consists of 8 modules [14], which covers six cognitive and social biases (attribution biases, jumping to conclusions, belief inflexibility, overconfidence in errors, the theory of mind deficits, and depressive cognitive schemata). Each session lasted about 60 min, and a gift worth $5 was given to each patient in the intervention group after each session, the whole course lasted for 8 weeks. In our program, only four patients withdrew at the first lesson due to family reasons.

Each MCT group comprised of about ten patients, and each intervention site had two groups. Due to community constraints, we made two minor adjustments: (1) the MCT was administered once a week, although the manual guideline was twice a week. Secondly, the MCT was delivered by a trained GP at a local community health center, while the manual recommended psychologists, psychiatrists, psychiatric nurses, and occupational therapists in the institution. None of the GPs had ever attended formal training in CBT. We, therefore, designed a four-stage training plan. The first-stage was concept understanding and the underlying theory of MCT, the second-stage was MCT manual studying, the third-stage was trail lectures, and the fourth-stage was the seminar and feedback. The GP-training course lasted for 1 month. The training was conducted by an experienced psychiatrist.

### Control group

The control group received a standard CBR for mental illness patients (see http://www.gov.cn/gongbao/content/2018/content_5338247.htm) for 8 weeks. In the first week, GPs together with patients and their respective families developed an individualized rehabilitation plan and a follow up plan conducted once during the project in the form of a phone call or home visits. The rehabilitation plan in CBR consisted of six aspects: medication training, relapse identification, physical management, life skills training, social skills training, and occupational rehabilitation training etc. Table 1 shows a comparison between MCT and CBR.

**Table 1:** Comparison of interventions

	MCT	CBR
Method	group activities	phone call, home visit, lessons
Frequency	once a week	once a quarter year
Duration	60 min	around 30 min
intervention content	MCT courses	rehabilitation counseling

### Assessments

Assessments were conducted at baseline and at week 9. All ratings followed semi-structured interviews. To ensure correspondence, baseline interviews were administered by the same assessor for each patient while post-treatment assessment was performed by a different assessor. The two raters were project-independent psychiatrists from Ningbo Mental Health Center.

#### Psychopathological assessment

The primary target of symptom severity of delusion was assessed with the Positive and Negative Syndrome Scale (PANSS) [39], which is sensitive to the change of symptoms [40]. Since we primarily targeted delusions, to be in line with previous research [16], we computed a sub-score of delusion (sum of the following items: P1 delusions, P5 grandiosity, P6 suspiciousness/persecution), as one of the significant outcome parameters.

As items in PANSS are highly condensed, we adopted Psychotic Symptom Rating Scales (PSYRATS) [41], which consist of two subscales of hallucinations and delusions, to measure possible dissociations across different aspects of positive symptoms and the severity of the syndrome. The PSYRATS had yielded good inter-rater, re-test reliability and validity [42].

#### Quality of life assessment

To measure the quality of life of patients, the Schizophrenia Quality of Life Scale (SQLS) [7], which provides estimates for the aspects of psychosocial, motivation and energy, symptoms, and side effects, was administered as the secondary outcome. The internal consistency reliability and construct validity was satisfactory [7]. In the SQLS assessment, the higher the scale, the worse the quality of life.

### Statistical analysis

We conducted per-protocol (pp) analyses. All statistical analyses were performed using SPSS 20.0. The patient’s baseline variables were compared between groups using independent t-tests or Chi-square tests. The repeated measure analysis of variance (ANOVA) was conducted to evaluate pre- and post-treatment effectiveness. The ANCOVA was applied to compare the effectiveness at post-treatment, with controlling for pre-treatment scores. Effect sizes were estimated using the partial eta squared (η^2^), the cut-off points of which were: small = 0.0099, medium = 0.0588, and large = 0.1379 [43]. All *P*-values are two-tailed, and *P* < 0.05 is considered statistically significant.

Multiple linear regression analyses (Stepwise) were performed to identify the factors that may independently contribute to quality of life. The scale change (post - pre) of three each aspect of SQLS was the dependent variable. We included group (intervention or control), sex, age, gender, age of onset, length of schizophrenia, years of formal education, marital status, length of illness, medication regimen, and improvement of several variables (PANSS core delusions, PANSS positive, PANSS negative, PANSS general, PSYRATS delusions, PSYRATS hallucinations, PSYRATS total) as dependent variables. To avoid co-linearity, we used tolerance to measure the strength of linear relationships (0.6 or above was acceptable). The 1-sample Kolmogorov-Smirnov test was performed to check the normality of the distributions for continuous variables.

## Results

### Baseline characteristics

Baseline characteristics for background and related psychopathological variables are shown in Table 2. There were no significant differences between the intervention and control groups. There were no significant differences in the percentage of patients who maintained the baseline medication plan between the two groups (86.20% vs. 93.55%, χ^2^ = 1.795, *p* = 0.181). Adherence was defined as the proportion of days the patients took their medication as prescribed in a month. A ratio of ≥90% was considered as regular. There was no significant difference between the two groups (79.31% vs. 67.74%, χ^2^ = 0.036, *p* = 0.849). Both groups had a chronic duration of schizophrenia (22.69 ± 12.05 vs. 29.55 ± 11.37, t = 0.217, *p* = 0.829), and a low level of education (6.32 ± 2.87 vs. 6.74 ± 2.46, t = 0.6.6, *p* = 0.547).

**Table 2:** Baseline characteristics

	MCT + CBR(*n* = 58)	CBR(*n* = 62)	χ^2^ / t	p
Age	55.28 (9.51)	52.90 (12.14)	0.839	0.405
Age of onset	32.59 (12.05)	29.55 (11.37)	1.004	0.319
Length of schizophrenia (years)	22.69 (12.02)	23.35 (12.70)	0.217	0.829
Years of formal education	6.32 (2.87)	6.74 (2.46)	0.606	0.547
Gender (male/female)	24/34	24/38	0.089	0.765
Marriage (Married/others)	34/24	36/26	2.427	0.489
Medication regimen (maintenance)	86.20%	93.55%	1.795	0.181
Taking adherence (regular)	79.31%	67.74%	0.036	0.849
Family history of Schizophrenia	10.34%	19.35%	1.908	0.167
Suffering from chronic diseases	34.48%	41.94%	0.704	0.401

### Outcomes

For PANSS, most of the scale assessments in the intervention group were significantly decreased at post-treatment as compared to pre-treatment, except for PANSS P5. However, in the control group, only PANSS P6, PANSS positive, PANSS negative, PANSS general, and PANSS total were significantly decreased at post-treatment.

After controlling for pre-treatment scores, a significant difference was found between the two groups in post-treatment period in terms of PANSS P6 (F (1,118) = 12.682, *p* = 0.001, η^2^_partial_ = 0.182), PANSS core delusions (F (1,118) = 9.13, *p* = 0.004, η^2^_partial_ = 0.138), PANSS positive syndrome (F (1,118) = 6.64, *p* = 0.013, η^2^_partial_ = 0.104), PANSS negative (F (1,118) = 6.51, p = 0.013, η^2^_partial_ = 0.102), PANSS general (F (1,118) = 12.039, p = 0.001, η^2^_partial_ = 0.174), and PANSS total (F (1,118) = 11.46, p = 0.001, η^2^_partial_ = 0.167).

For PSYRATS, significant effects were obtained for all three scales of the intervention group at post-treatment as compared to pre-treatment. In the control group, only PSYRATS total was significant at post-treatment compared with pre-treatment.

After controlling for pre-treatment scores, a significant difference was noted in the post-treatment period between the two groups in terms of PSYRATS delusions (F (1,118) = 4.43, *p* = 0.04, η^2^_partial_ = 0.072), and PSYRATS total (F (1,118) = 4.32, *p* = 0.042, η^2^_partial_ = 0.071).

In the intervention group, the three SQLS scales at post-treatment were different from those of pre-treatment. The control group was only significantly improved on SQLS symptoms and side-effects. While controlling for the pre-treatment scores, a significant difference was found in the two groups in the post-treatment in terms of the SQLS psychosocial (F (1,118) = 6.55, *p* = 0.013, η^2^_partial_ = 0.103). See Table 3.

**Table 3:** Group differences on measures of PSYRATS, PANSS, SQLS

Variable	MCT + CBR(n = 58)			CBR(n = 62)			Post-treatment comparisons Between-group (ANCOVAs controlling for baseline scores)
	Pre-treatment	Post-treatment	pre - post	Pre-treatment	Post-treatment	pre - post	
PSYRATS delusions	8.45 (5.35)	6.61 (3.77) *	1.84	8.00 (5.41)	7.65 (5.08)	0.35	F (1,118) = 4.43, p = 0.04, η^2^_partial_ = 0.072
PSYRATS hallucinations	14.03 (10.45)	7.90 (9.17) ***	6.13	13.10 (9.75)	10.86 (9.93)	2.24	F (1,118) = 3.15, *p* = 0.081, η^2^_partial_ = 0.052
PSYRATS total	22.48 (14.51)	14.52 (11.97) **	7.96	21.10 (13.70)	18.52 (13.28) *	2.58	F (1,118) = 4.32, p = 0.042, η^2^_partial_ = 0.071
PANSS P1 (delusions)	2.74 (1.03)	2.48 (0.89) **	0.26	2.66 (1.26)	2.55 (1.06)	0.11	F (1,118) = 1.77, *p* = 0.189, η^2^_partial_ = 0.030
PANSS P5 (grandiosity)	2.00 (1.10)	1.74 (0.77)	0.26	1.79 (1.05)	1.86 (1.06)	−0.07	F (1,118) = 2.34, *p* = 0.132, η^2^_partial_ = 0.039
PANSS P6 (suspiciousness)	2.55 (1.63)	1.58 (0.81) ***	0.97	2.48 (1.50)	2.24 (1.15) **	0.24	F (1,118) = 12.682, p = 0.001, η^2^_partial_ = 0.182
PANSS core delusions	7.29 (3.21)	5.81 (1.80) ***	1.48	6.93 (3.37)	6.66 (2.94)	0.27	F (1,118) = 9.13, p = 0.004, η^2^_partial_ = 0.138
PANSS positive	16.87 (6.23)	13.61 (3.81) ****	3.26	15.28 (5.89)	14.62 (5.27) *	0.66	F (1,118) = 6.64, p = 0.013, η^2^_partial_ = 0.104
PANSS negative	16.67 (4.45)	14.10 (3.17) **	6.57	18.83 (5.57)	17.24 (4.63) *	1.59	F (1,118) = 6.51, p = 0.013, η2partial = 0.102
PANSS general	38.16 (7.94)	33.58 (5.34) ****	4.58	39.59 (9.06)	37.69 (6.84) **	1.9	F (1,118) = 12.039, p = 0.001, η2partial = 0.174
PANSS total score	71.71 (2.72)	61.29 (9.48) ****	10.42	73.69 (17.53)	69.55 (14.34) ****	4.14	F (1,118) = 11.46, p = 0.001, η^2^_partial_ = 0.167
SQLS psychosocial	27.47 (12.32)	24.3 (10.29) *	3.17	26.63 (11.96)	27.82 (10.55)	−1.19	F (1,118) = 6.55, p = 0.013, η^2^_partial_ = 0.103
SQLS motivation and energy	51.61 (14.31)	47.93 (13.42) *	3.68	59.85 (12.69)	58.00 (12.06)	1.85	F (1,118) = 3.64, *p* = 0.062, η^2^_partial_ = 0.06
SQLS symptoms and side-effects	22.28 (12.80)	18.45 (10.56) *	3.83	22.41 (15.98)	20.37 (13.17) *	2.04	F (1,118) = 1.57, *p* = 0.216, η^2^_partial_ = 0.027

*Abbreviations: MCT* Metacognitive Training, *CBR* Community-based rehabilitation, *PANSS* Positive and Negative Syndrome Scale, *PSYRATS* Psychotic Symptom Rating Scales, *SQLS* Quality of Life Scale

Significant difference from zero: *p ≤ 0.05, **p ≤ 0.01, ***p ≤ 0.005, ****p ≤ 0.001

The results of the multiple regression analyses conducted to identify the contributors to three aspects of SQLS are shown in Table 4. Improvement of SQLS psychosocial was independently associated with low PSYRATS hallucinations and low PANSS core delusions. Improvement of motivation and energy was independently associated with low PANSS core delusions. SQLS symptoms and side-effects were independently associated with low PSYRATS total.

**Table 4:** Variables independently associated with the three aspect of SQLS in multiple linear regression analysis

Dependent variable		Beta	***T***	***P***	95%Confidence interval
SQLS psychosocial	PSYRATS hallucinations	0.305	3.089	0.003	−0.107 to 0.503
	PANSS core delusions	1.279	2.857	0.006	0.383 to 2.175
SQLS motivation and energy	PANSS core delusions	0.938	2.051	0.045	0.022 to 1.854
SQLS symptoms and side-effects	PSYRATS total	0.317	4.347	< 0.001	0.171 to 0.463

## Discussion

This is the first trial conducted to examine the efficacy of MCT in community rehabilitation for patients with schizophrenia. The results confirmed our assumption that MCT can improve positive symptoms, particularly delusion symptoms [28].

By comparing several scores (PSYRATS total, PANSS positive, PANSS negative, PANSS general, and PANSS total) between pre-treatment and post-treatment, we found that the scores of both groups decreased to varying degrees, meaning, the overall psychiatric symptoms of both groups improved. It may be translated that the method taken by both groups was effective. While as in the intervention group, almost all the scales showed significant improved after the intervention, it indicated that the conventional CBR maybe effective to some extent but was not as comprehensive as CBR + MCT.

To further analyze the effect of MCT, we conducted ANCOVAs and controlled the baseline scores to compare post-treatment effects between the two groups. The PANSS core delusions showed significant improvement in intervention group when compared to the control group at post-treatment. Since the PANSS core delusions are the sum of P1, P5, and P6, we also computed the scores of each. Given that there was no significant difference in P1 and P5, the difference in PANSS core delusions may be mainly caused by P6 (suspiciousness/persecution). Some study indicated that the high risk of violence may sometimes be attributed to the delusions [44], among which patients are more likely to act on persecutory delusions [45]. Since our results show that MCT may improve the patients’ suspiciousness/persecution symptoms, we consider that MCT may decrease the risk of community violence.

The PANSS positive syndrome, PANSS negative, PANSS general, and the PANSS total scores were significantly different between the two groups at post-treatment. This showed that the MCT comprehensively improved the patient’s symptoms. A previous study [30] suggested that MCT can reduce positive symptoms in schizophrenia patients, and negative symptoms when combined with CBR at the community level. As for the reason why negative symptoms was improved, a study indicated that responses of patients to persecutory delusions may be associated with negative symptoms [45], herein, the MCT improved the persecutory delusions of the intervention group and thus affected the PANSS negative scale.

The PSYRATS scale enables a more detailed evaluation of delusions. As reported in a meta-analysis [28, 29], results of ANCOVAs confirmed that the intervention group showed significant improvements in the PSYRATS delusions and the PSYRATS total scale as compared to the control group.

The QOL of patients in the intervention group improved at post-treatment in all three dimensions: psychosocial, motivation and energy, and symptoms/side effects. The control group showed significant changes only in the symptom/side effect dimension at post-treatment. The improvement of symptoms/side effects domain maybe the result of the medication [9] and deinstitutionalization [46]. Since the percentage of patients who followed the baseline medication plan was not different between the two groups, we assumed that the improvement in symptoms/side effects was mainly because of the community intervention, either MCT or CRB.

Results of ANCOVAs analysis showed that the effect of the psychosocial dimension in SQLS of the intervention group was superior to that of the control group. In line with prior findings [24], MCT improved the quality of life, particularly its social aspects. The psychosocial dimension of SQLS mainly covers the patient’s emotional problems and attitudes towards the society and the future [7]. Thus, MCT may help patients to better control their emotions and build proper expectations for the future, especially their psychological well-being and social relationships [24]. Multiple regression analysis also showed that improvement of SQLS was independently associated with low positive psychotic symptoms. From this perspective, while MCT relieved positive symptoms, it may indirectly improve the patient’s quality of life. Previous study [47] reported that both positive and negative symptoms influence the quality of life of patients, our findings support this conclusion and again emphasize the importance of positive symptoms in determining the quality of life of schizophrenia patients in community.

In summary, our results show that MCT performed in the community, described as a hybrid of cognitive-behavioral therapy and psychoeducation [48], is effective. In China, CBR for patients with mental illness was mainly based on basic public health packages [6]. The services focus more on disease monitoring rather than community rehabilitation. Therefore, interventions that are relatively simple, exciting, and effective at using local community resources [49] are recommended.

### Limitations

Community trails are usually characterized by limited resources [50]. However, flexibility in the program delivery might have influenced our findings. In our study, recruiters and patients were familiar with each other, making it easier to recruit patients and help patients adhere to the program. Good relationships may be a positive contributor to QOL in patients with prolonged illness [51], which would have a positive influence on the result. In addition, to increase patient participation in nearby MCT courses, we set up three intervention points, which may cause selection bias to some degree. Although we developed a reinforcement plan for the intervention group, some factors may affect the motivation for treatment, and may lead to higher compliance in the intervention group patient compared to the control group.

Second, the MCT courses used several Western characters and stories, which may affect understanding, and thus, influenced its effects. We shall conduct further localization research based on cultural characteristics, historical, and language characteristics.

Third, the study was limited by the shorter patient follow up. However, one of our major purposes was to find an effective intervention method for future implementation in the community. It should be noted that sustained effects for 6 months [28] and 6 months to 3 years were controversially reported [15, 52]. In the future, we may roll out regular MCT courses at the community level. The long-term effect and the effects of patients repeatedly attending these courses will be evaluated to find a more cost-benefit arrangement in the community. The developers of MCT are also exploring the possibility of online teaching [48]. Efforts should also be made to shorten the course (to reduce cost) [18].

Finally, future studies with a larger sample should take into consideration comorbid symptoms as moderator variables. Based on previous studies [53] it is expected that those with high on social anxiety and low on self-esteem will benefit most from the MCT intervention. We also suggest that the MCT app [48] should be translated into Chinese to augment its long-term effects especially in those with memory problems, for example due to comorbid neurological problems.

## Conclusions

Our results show that MCT can be adopted in community rehabilitation for patients with schizophrenia.

## Data Availability

Data and materials of this study are available from the corresponding author upon reasonable request.

## Abbreviations

CBR: Community-based rehabilitation
MCT: Metacognitive Training
GP: general practitioner
PANSS: Positive and Negative Syndrome Scale
PSYRATS: Psychotic Symptom Rating Scales
SQLS: Quality of Life Scale
MHD: mental health and development
CDC: Center for Disease Control and Prevention
EHS: Electronic Healthcare System
